# Dianxianning improved amyloid β-induced pathological characteristics partially through DAF-2/DAF-16 insulin like pathway in transgenic ***C. elegans***

**DOI:** 10.1038/s41598-017-11628-9

**Published:** 2017-09-12

**Authors:** Dejuan Zhi, Dong Wang, Wenqi Yang, Ziyun Duan, Shuqian Zhu, Juan Dong, Na Wang, Ningbo Wang, Dongqing Fei, Zhanxin Zhang, Xin Wang, Meizhu Wang, Hongyu Li

**Affiliations:** Gansu high throughput screening and creation center for health products, School of Pharmacy, Lanzhou University, Lanzhou, P.R. China

## Abstract

Dianxianning (DXN) is a traditional Chinese formula, and has been approved in China for treating epilepsy since 1996. Here anti-Alzheimer’s disease activity of DXN has been reported. DXN improved AD-like symptoms of paralysis and 5-HT sensitivity of transgenic Aβ_1-42_ *C. elegans*. In worms, DXN significantly increased Aβ monomers and decreased the toxic Aβ oligomers, thus reducing Aβ toxicity. DXN significantly suppressed the expression of *hsp-16.2* induced by juglone, and up-regulated *sod-3* expression. These results indicated that DXN increased stress resistance and protected *C. elegans* against oxidative stress. Furthermore, DXN could significantly promote DAF-16 nuclear translocation, but it did not activate SKN-1. The inhibitory effect of DXN on the Aβ toxicity was significantly reverted by *daf-16* RNAi, rather than *skn-1* RNAi or *hsf-1* RNAi. These results indicated that DAF-16 is at least partially required for the anti-AD effect of DXN. In conclusion, DXN improved Aβ-induced pathological characteristics partially through DAF-2/DAF-16 insulin like pathway in transgenic worms. Together with our data obtained by Morris water maze test, the results showed that DXN markedly ameliorated cognitive performance impairment induced by scopolamine in mice. All the results support that DXN is a potential drug candidate to treat Alzheimer’s diseases.

## Introduction

Alzheimer’s disease is a progressive neurodegenerative disorder which is becoming more prevalent in ageing populations worldwide. It is neuropathologically characterized by extensive neuronal loss and the presence of neurofibrillary tangles and senile plaques^1^. Nowadays over 46 million people suffer from AD in the world, and it is estimated to increase to 131.5 million by 2050^2^. So far, there are only five FDA-approved anti-AD drugs on the market. Unfortunately, these drugs can only delay the onset of dementia, and none of them can halt or reverse the disease. Those medications for treating AD have never met the medical need, it is urgent to find effective and safe drugs against AD.

A lot of evidence have shown that the pathological hallmarks of AD are included Aβ aggregation and deposition with senile plaque appearance, tau hyper phosphorylation with tangle formation, which destroy synapses, induce brain inflammation, eventually lead neuronal death and severe brain shrinkage^3^. Among them, the Aβ is generated in brain from the amyloid precursor protein (APP) after sequential proteolytic cleavage by β-secretase and γ-secretase enzymes^4^. Under normal conditions, the produced Aβ is quickly removed from the brain. However, the mutations that increase Aβ production and the factors that decrease Aβ clearance or enhance Aβ aggregation will lead to Aβ self-aggregates into assemblies ranging from oligomers to protofibrils, fibrils and amyloid plaques^5^. Aβ cascade hypothesis of AD is widely accepted^6^. Accordingly, the anti-AD therapies primarily aim to lower the level of Aβ generation, aggregation or accumulation in the brain of AD patients. However, many of such drug candidates were demonstrated to manifest only limited benefit in AD patients. The reason is mainly explained by that other complex pathogenic cascades of related Aβ induced toxicity, are not affected, thereby the overall effects of those therapies being negligible^4, 7^. Obviously, more factors of the pathogenic cascades need to be considered in the process of anti-AD drug discovery.

Previous studies have indicated that AD patients have an increased risk of developing epileptic seizures, and the risk of epileptic activity is highest in those patients with early-onset dementia who over-express amyloid precursor protein (APP) and Aβ^6^. Epidemiological data indicates that patients with AD and seizure disorders have greater cognitive impairment, faster progression of symptoms, and more severe neuronal loss at autopsy than those without seizures^8^. There is a close relationship between epilepsy and AD, and Aβ has been identified to be the link between these two disorders^9^. In fact, antiepileptic drugs have been involved in preclinical studies or clinical trials for treating AD. Levetiracetam can suppress neuronal network dysfunction and reverse AD damage and cognitive impairment in mice^6^. Lamotrigine can attenuate deficits in synaptic plasticity and accumulation of amyloid plaques in APP/PS1 transgenic mice and effectively promote their learning memory behavior^10^. Valproic acid can inhibit Aβ generation, neuritic plaque production, ameliorate cognitive performance in AD mice, but it is unlikely to affect patient cognitive function^11, 12^. Carbamazepine and phenytoin can even impair patient cognitive function^11^. Anyhow, repurposing antiepileptic drugs is still available to discover potential anti-AD drug candidates.

The Dianxian Ning (DXN) has been approved in China Food and Drug Administration as an antiepileptic drug since 1996. DXN consists of eight Chinese medicine herbs, namely *Valeriana jatamansi Rhizoma et Radix, Rhizoma Acori tatarinowii, Ramulus Uncariae cum Uncis, Semen Pharbitidis, Semen Euphorbiae, Radix et Rhizoma Valeriana officinalis, Rhizoma et Radix Nardostachys*, and menthol crystal^13^. According to the theory of traditional Chinese medicine (TCM), DXN can clinically eliminate phlegm for resuscitation, stop wind, and calm the spirit^13^. In fact, TCM similarly function on a multi-targeted manner to treat the symptoms. Previous work reported that *Valeriana jatamansi Rhizoma et Radix* possesses anti-inflammatory, anti-oxidant activities^14, 15^. *Rhizoma Acori Tatarinowii* can exert neuroprotective action to battle against AD^1^. *Ramulus Uncariae cum Uncis* can inhibit Aβ formation, and compounds extracted from this herb have neuroprotective activity^16, 17^. However, how those eight herb components in one formula of DXN work together, and whether DXN can act as a complete formula or to be reduced, or as a resource of any effective compound for treating AD remains unclear.

*Caenorhabditis elegans* is an inexpensive tool to be widely used to evaluate anti-AD drug candidates based on Aβ hypothesis^18^. In this study, we investigated the effect of DXN on Aβ-induced injury using transgenic *C. elegans* model which exhibits several pathological behaviors due to Aβ toxicity. We also examined whether DXN can improve cognitive performance impairments induced by scopolamine in mice by Morris water maze test. Finally, we explored the underlying mechanism that DXN exerted its possible anti-AD action. Our results provide evidences for that DXN as a traditional Chinese anti-epileptic medicine is likely to be a potential drug candidate to fight against AD. Also, our results provide clues to introduce anti-epileptic TCM as potential drug candidate discovery resources for treating AD.

## Results

### DXN ameliorated AD-like symptoms induced by Aβ_1-42_ expression

**Figure 1:**
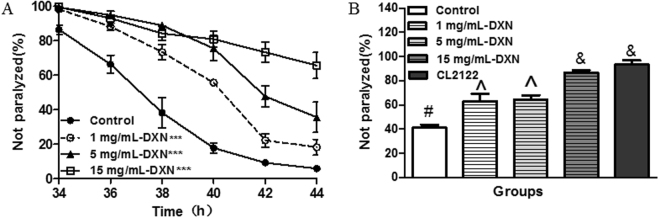
The effects of DXN on AD-like symptoms in Aβ transgenic *C. elegans*. (**A**) DXN delayed AD worm paralysis induced by Aβ over-expression in muscle tissues in *smg-1* (cc546)I temperature sensitive Aβ strain CL4176. Data are the average of three replicates with about 180 worms in each group. ***Indicated that there was significant difference between treatment group and control group at P < 0.001. (**B**) DXN ameliorated 5-HT hypersensitivity induced by Aβ over-expression in nerve cells in *snb-1*/Aβ1-42 strain CL2355. Worm strain CL2122 without Aβ expression in nerve cells was used as a transgenic control. Data are the average of three replicates with about 90 worms in each group. There is significant difference among these groups when symbols are different (P < 0.05).

To determine whether the DXN can protect against the toxicity induced by Aβ_1-42_ expression, transgenic *C. elegans smg-1* (cc546)I temperature sensitive Aβ strain CL4176 was used, and human Aβ_1-42_ peptide has been transferred into worms and controlled by the promoter of *myo-3*, thus the over expression of Aβ_1-42_ in the muscle cells can induce AD-like symptom of Aβ-dependent paralysis. We found that DXN significantly delayed worm paralysis in a dose-dependent manner (Fig. 1A).

Serotonin (5-HT) is an important neurotransmitter that regulates worm behaviors of locomotion, egg-laying, olfactory learning and mating^19–21^. Exogenous 5-HT can inhibit *C. elegans* locomotion, leading active worm into paralysis. *C. elegans snb-1*/Aβ_1-42_ strain CL2355 shows serotonin hypersensitivity when Aβ are over-expressed in nerve system^22, 23^. Here we investigated whether DXN treatment could reverse serotonin hypersensitivity induced by the toxicity of Aβ to nerve cells in the transgenic *snb-1*/Aβ_1-42_ worms. Our results showed that *snb-1*/Aβ_1-42_ worms were hypersensitive to exogenous 5-HT, but CL2122 without Aβ expression in nerve cells were not. DXN significantly alleviated the paralysis symptom of hypersensitive response to 5-HT in a dose-dependent manner (Fig. 1B).

**Figure 2:**
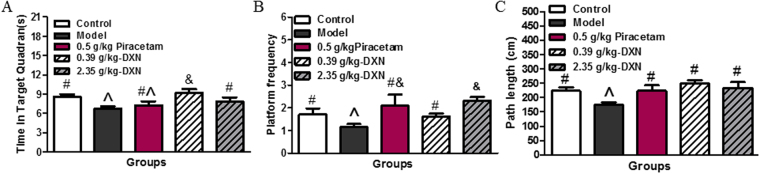
The effects of DXN on cognitive impairment in mice induced by scopolamine in Morris water maze test (MWM). (**A**) The mean time in probe trial of MWM. (**B**) The mean platform frequency in probe trial of MWM. (**C**) The swimming path length in probe trial of MWM. Data are the average of 14 animals in each group. There is significant difference among these groups when symbols are different (P < 0.05).

Scopolamine is an anti-cholinergic agent to induce aspects of the memory loss and cognitive impairment, which resemble the hallmark symptoms observed in AD associated with cholinergic dysfunction at least on some degree. The scopolamine model has been widely used for screening possible AD symptom therapeutic agents to improve cognitive performances^24^. In present work, this model was used to evaluate the effects of DXN on learning and memory impairment by the Morris water maze test. Compared with the normal control group, the memory training results indicated that the mean escape latencies were significantly lengthened in scopolamine treated mice on the 5^th^ day of the experiment. DXN improved learning and memory performance of mice as piracetam (Table 1). In probe test, the swimming time in the target quadrant of DXN treated animals significantly lengthened in contrast to the model group (Fig. 2A). The platform frequency remarkably increased in DXN and piracetam treated groups (Fig. 2B). Compared to model group, the length of swimming path between drug treatment groups and normal control group were not significant in probe test (Fig. 2C). These results suggested that DXN indeed promoted learning and memory performance in scopolamine treated mice.

**Table 1:** Comparison of average escape latency time for each group in the hidden platform trial.

Treatment	Escape latency time/second				
	1^st^ day	2^nd^ day	3^rd^ day	4^th^ day	5^th^ day
Control	38.37 ± 3.25	33.60 ± 4.45	29.76 ± 2.96	21.36 ± 4.19	16.54 ± 2.72^**#**^
Model	45.06 ± 1.90	42.64 ± 1.87	32.68 ± 3.93	33.28 ± 2.72	34.18 ± 2.68^**^**^
0.5 g/kg Piracetam	41.01 ± 2.62	33.02 ± 3.04	29.83 ± 2.79	28.06 ± 3.33	21.07 ± 2.61^**#**&^
0.39 g/kg-DXN	35.77 ± 3.65	31.08 ± 3.63	33.11 ± 3.51	27.61 ± 3.63	25.75 ± 3.22^&^
2.35 g/kg-DXN	43.86 ± 3.29	35.43 ± 3.08	34.29 ± 3.66	27.87 ± 3.05	20.71 ± 3.14^**#**&^

Data are the average of 14 animals in each group. There is significant difference among these groups when symbols are different (*P* < 0.05).

### DXN exerted anti-AD like action by its principal component *Valeriana jatamansi Rhizoma et Radix*

DXN is a kind of traditional Chinese medicine, and it is composed of eight herbs. Previous works have suggested that three herbs of *Valerianae jatamansi Rhizoma et Radix* (Vj), *Rhizoma Acori tatarinowii* (Ra) and *Ramulus Uncariae cum Uncis* (Ru) in DXN have anti-AD activity^1, 14–17^. In present work, Vj, Ra and Ru at an equivalent dose in DXN were firstly used to tested whether they can ameliorate AD-like symptom of delaying paralysis in worms. We further tried to investigate that whether DXN can be reduced to one or more herbs which can function as the principal component, and finally some effective compounds based anti-Aβ therapy for treating AD can be extracted from the right principal herbs.

**Figure 3:**
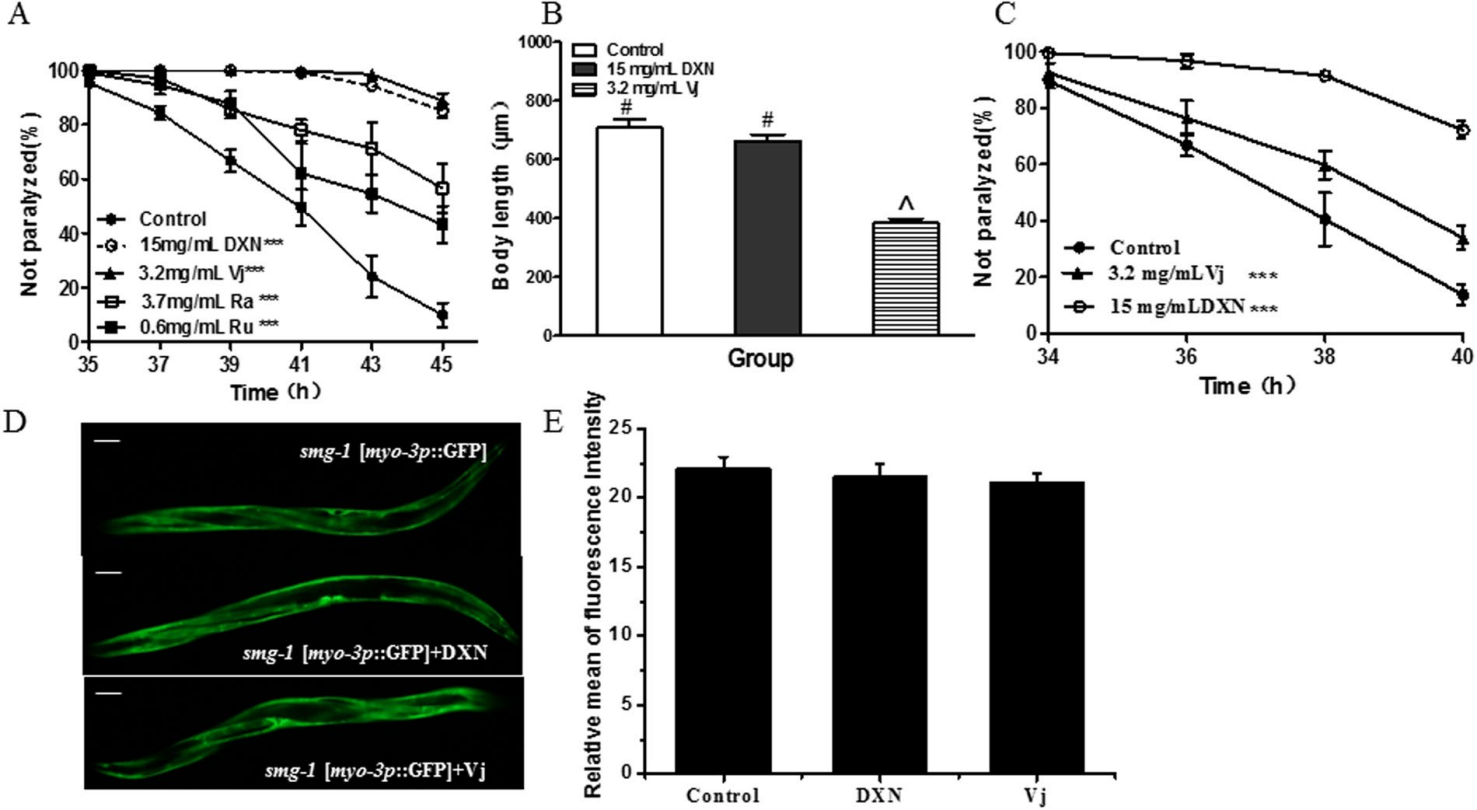
DXN and its principal component *Valeriana jatamansi* (Vj) delayed worm paralysis similarly, but it could not be reduced due to its safety. (**A**) Paralysis assays for the temperature sensitive Aβ strain CL4176 treated with DXN or other herbs of Ra, Ru and Vj. Data are the average of three replicates with about 180 worms in each group. ***Indicated that there was significant difference between treatment group and control group at P < 0.001. (**B**) The effects of DXN and Vj on the body length of AD worms tested. The concentration of herb of Ra, Ru and Vj was equivalent to each of them containing in DXN. There is significant difference among these groups when symbols are different (P < 0.05). (**C**) Paralysis assays for the temperature sensitive Aβ strain CL4176 treated with DXN from the onset of paralysis. Data are the average of three replicates with about 180 worms in each group. ***Indicated that there was significant difference between treatment group and control group at P < 0.001. (**D**) The effect of DXN on Vj on GFP expression in *smg-1*[*myo-3p*::GFP] worms. (**E**) The relative mean fluorescence intensity of worms treated with or without drugs. Data are the average of three replicates with about 90 worms in each group. The scale bar was 40 μm.

The results showed that Vj delayed worm paralysis as DXN complete formula to a similar degree. Ra and Ru significantly alleviated the AD-like symptom in worms to a much lesser degree (Fig. 3A). These results supported that Vj was the principal component of DXN. It is deserved to notice that Vj alone also significantly shortened worm body length, but DXN did not (Fig. 3B). In contrast to drug treatment from eggs, worms were administrated with DXN or Vj when incubation temperature was upshifted to induce Aβ expression, and at that time animals were on the same development stage. The results showed that DXN and Vj did delay worm paralysis, but to a less degree (Fig. 3C). Of course, shorter dosing time and less DXN and Vj entry into worms led the decline in anti-AD activity of DXN and Vj for delaying paralysis. Anyway, the effect of Vj largely decreased than that of DXN did (Fig. 3A,C). Taken together, those results supported that complete DXN was a rational formula as it is and it should not be reduced. Additionally, transgenic worm *smg-1*[*myo-3*::GFP] was further used to test whether DXN treatment non-specifically affected gene expressions, the results showed that neither DXN nor Vj treatment altered exogenous *gfp* gene expression (Fig. 3D,E), suggesting that DXN and Vj treatment possible inhibiting non-specific protein expression could be rule out.

**Figure 4:**
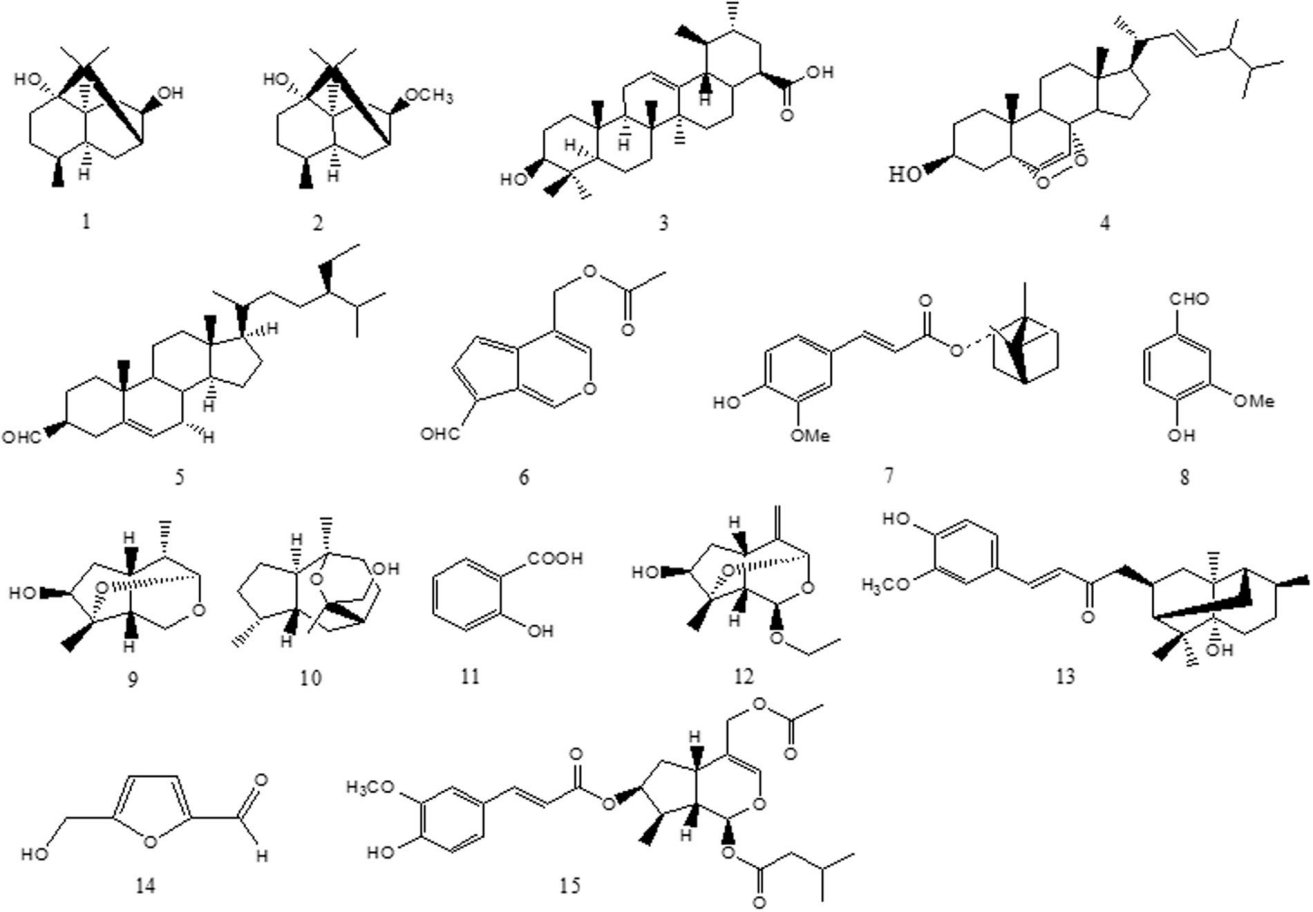
Compounds of 1–15 extracted from *Valeriana jatamansi* 1: 8-hydroxyl-pathcouli alcohol; 2: 8-acetoxyl-pathchouli alcohol; 3: Ursolic acid; 4: 3β- Hydroxy-5α,8α-epidioxyergosta-6,22-diene; 5: stigmast-5-ene-3β-ylformate; 6: baldrinal; 7: (-)-bornyl ferulate; 8: vanillin; 9: (3S, 4S, 5S, -7S, 8S, 9S)-3,8-ethoxy-7-dihydroxy-4,8- dimethylperhydro cyclopenta - [c] pyran; 10: valerol A; 11: salicylic acid ;12: (4β,8β)-8-ethoxy-3-methyl -10- methylen-2,9-dioxatricyclo[4.3.1.03,7]decan-4-ol; 13: Valeriananoids D; 14: 5- (hydroxymethyl) - 2-furfuraldehyde; 15: 8-methylvalepotriate.

**Figure 5:**
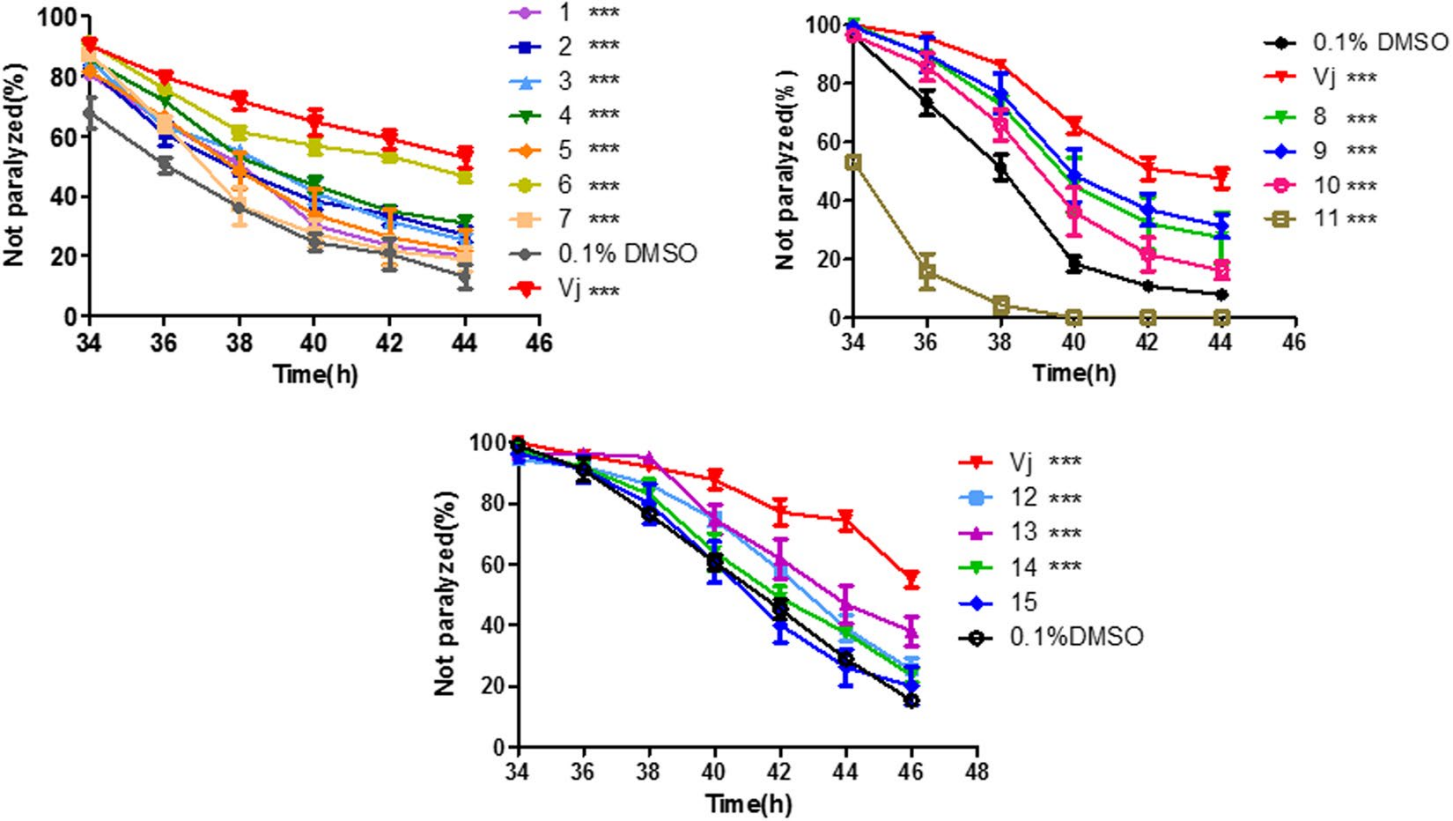
The effects of compound 1–15 extracted from DXN on AD-like symptoms in the temperature sensitive Aβ worms. Data are the average of three replicates with about 180 worms in each group. ***Indicated that there was significant difference between treatment group and control group at P < 0.001.

Iridoids and sesquiterpenoid derived from Vj has been reported to have neuroprotective activity^25^. Here, we further evaluated compound 1–15 extracted from Vj (Fig. 4), and treated worms with each compound at relative final concentration due to their different maximum saturated solubility in DMSO. In contrast to Vj, almost all of them delayed worm paralysis induced by over-expression of Aβ to a much lesser degree, except that 100 μM of compound 15 did not have any anti-AD activity, 250 μM of compound 11 significantly promoted worm paralysis (Fig. 5). It is deserved to notice that compound 6 is baldrinal, which is regarded as the marker substance of Vj and DXN, and its content is 0.930 mg per gram of DXN or Vj coarse powder. In Fig. 5, 160 µM baldrinal was at a dosage higher by 2.6-fold than that contained in Vj used. Previous work showed that an iridoid, 10-O-trans-p-coumaroylcatalpol at similar dose or less significantly inhibits α-synuclein aggregation in worms^26^. So that, it cannot be excluded that more active compound will be isolated from Vj in the future work. Because relative large amount of baldrinal is contained in Vj, its anti-AD activity is still dissecting in our lab. At present, it is reasonable to believe that DXN needs complete Vj.

### DXN reduced Aβ oligomers rather than amyloid deposits in transgenic *C. elegans*

**Figure 6:**
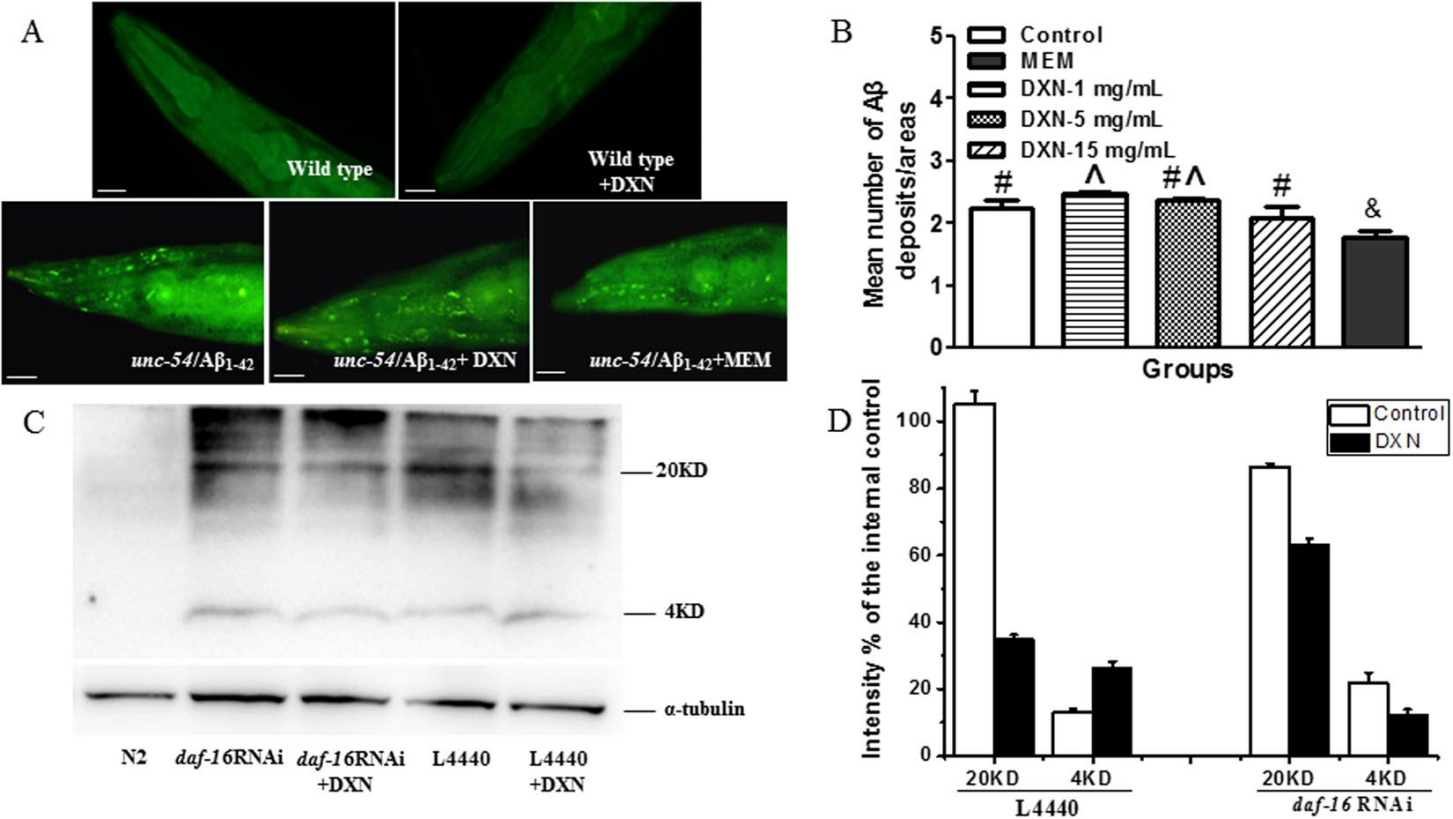
The effect of DXN on Aβ deposits or Aβ oligomers in AD worms. (**A**) ThS staining images of Aβ deposits in *unc-54*/human Aβ_1-42_ worms after treatment with or without 15 mg/mL DXN, and 10 mM memantine (MEM) was used as positive control. Wild type N2 was used as ThS Aβ-specific staining control. The scale bar was 20 μm. Data are the average of three replicates with about 24 worms in each group. (**B**) Quantification of ThS-positive particles in head region of *unc-54*/human Aβ_1-42_ worms. There is significant difference among these groups when symbols are different (P < 0.05). (**C**) Western blot of Aβ species in the temperature sensitive Aβ strain CL4176 treated with or without 15 mg/mL DXN. (**D**) Band intensity of 4 KD Aβ monomers or 20 KD oligomers in C were analyzed by Image J software. Data are the average of three replicates with about 400–500 worms in each group.

To explore whether the DXN affects the aggregation of the large Aβ species, unc-54/human Aβ_1-42_ worm strain CL2006 that constituently over expresses human Aβ_1-42_ in muscle cells was used. Although memantine is clinically used as a selected NMDA receptor antagonist, evidence recently indicates that it lowers the Aβ level and decreases Aβ plaques to support memantine may be a disease-modifying drug for treating AD^27–30^. Therefore, memantine has been used as a positive control. We found the DXN had no remarkable effect on the deposits of the Aβ_1-42_ peptide, suggesting that the DXN could not decrease the aggregation of the Aβ species significantly (Fig. 6A,B). As expected, memantine significantly lowered Aβ deposits (Fig. 6A,B). Considering the current evidence shows that soluble Aβ oligomers, rather than senile plaques (SPs) or Aβ fibrils, are the toxic species to link to neurodegeneration and cognitive decline^31, 32^, we performed the Western blotting analyses to test whether the DXN can reduce the toxic Aβ oligomers.

Primary antibody of 6E10 against Aβ was used in western blotting analysis, and the relative densities of Aβ monomer of 4 kDa band and higher molecular weight oligomer of 20 kDa band were quantified^22^. The result showed that DXN significantly reduced the level of Aβ oligomers and increased the level of Aβ monomers (Fig. 6C,D). It indicated that DXN could significantly change the Aβ oligomers with high neurotoxicity into Aβ monomers, which is much less toxic than Aβ oligomers.

### DXN delaying paralysis of AD worms was partially mediated by DAF-16 activation

**Figure 7:**
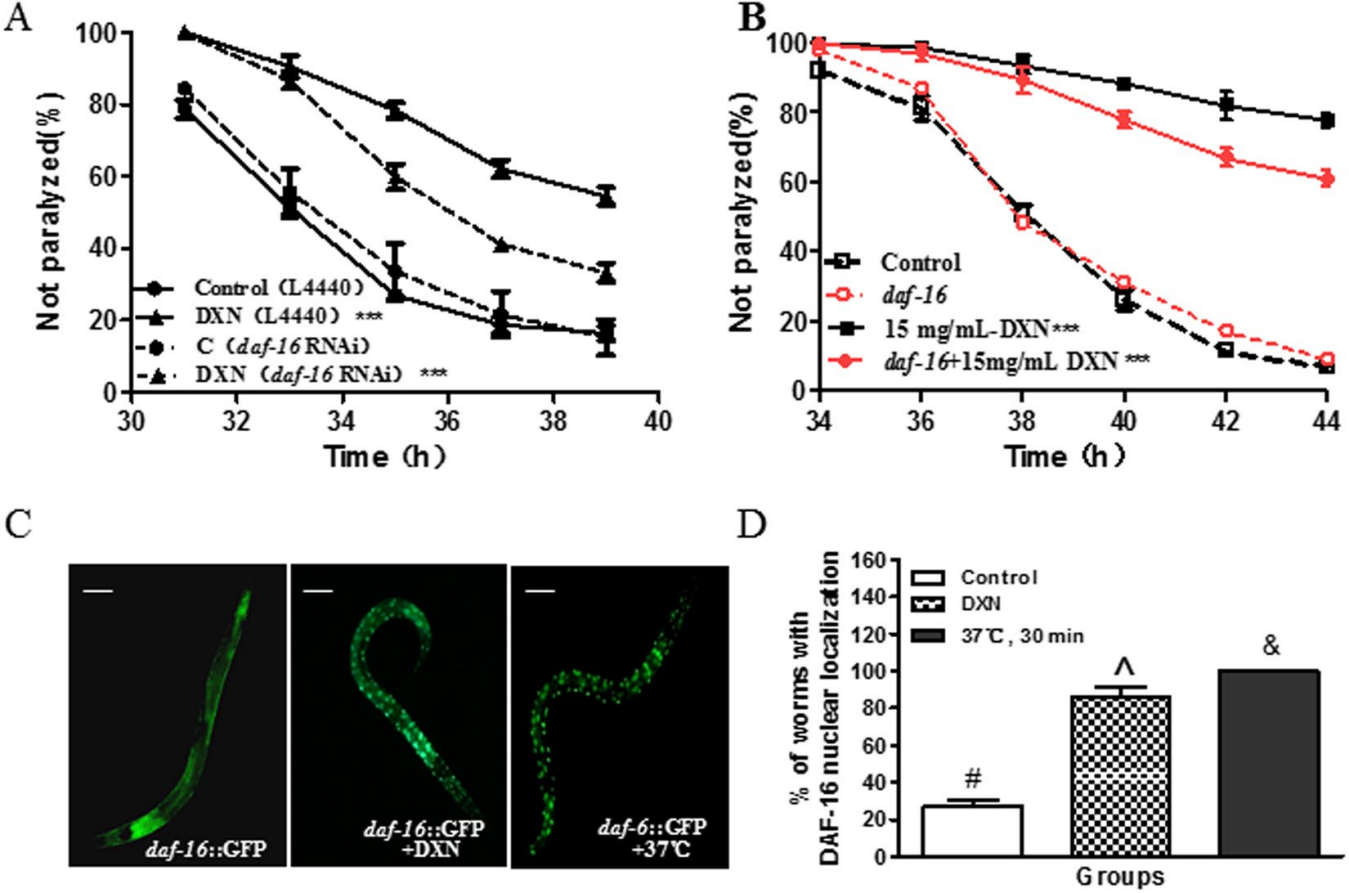
DXN inhibiting Aβ toxicity was at least partially mediated by DAF-16. (**A**) DXN inhibiting worm paralysis induced by Aβ over-expression was partially reverted by *daf-16* RNAi. (**B**) The effect of DXN was similar partially reverted by *daf-16* deletion. (**C**) P*daf-16*::*gfp* reporter was induced in worm strain TJ356 after treatment with 15 mg/mL DXN, and heat shock treatment at 37 °C for 15 min as a positive control. The scale bar was 80 μm. (**D**) Quantification of DAF-16 nuclear localization. In A and B, data are the average of three replicates with about 120-180 worms in each group. In C and D, animals were about 90 individuals. ***Indicated that there was significant difference between treatment group and control group at P < 0.001. There is significant difference among these groups when symbols are different (P < 0.05).

**Figure 8:**
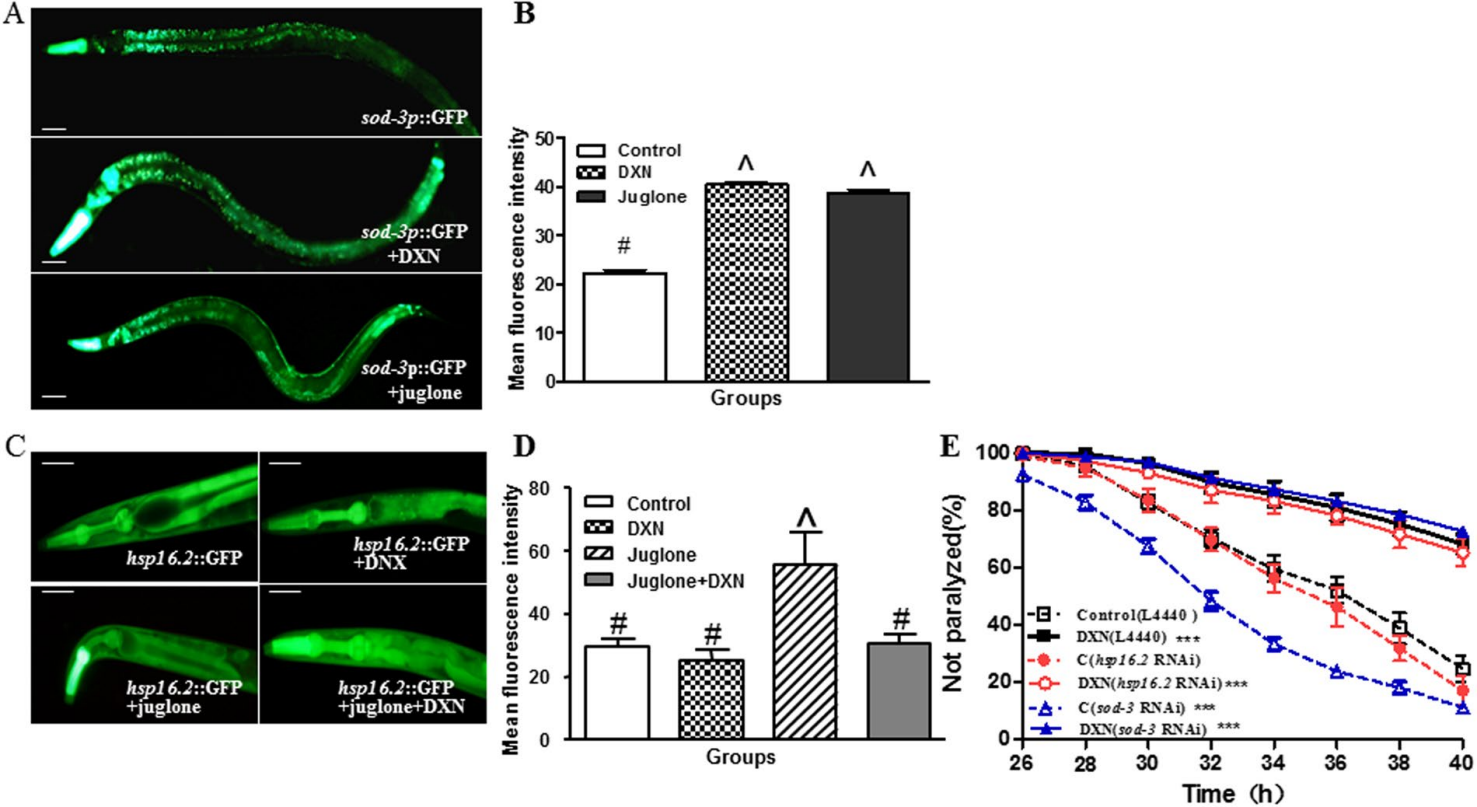
SOD-3 was required for DXN inhibiting Aβ toxicity. (**A**) *sod-3* expression was up-regulated after 15 mg/mL DXN treatment, and worms were treated with 20 mM juglone as positive control. The scale bar was 40 μm. (**B**) Quantification of *sod-3* expression. (**C**) 15 mg/mL DXN inhibited the expression of P*hsp-16.2*::GFP induced by 40 μM juglone, and worms treated with 20 mM juglone were used as positive control. The scale bar was 25 μm. (**D**) Quantification of the expression of *hsp-16.2*. In (**A**–**D**), data are the average of three replicates with about 90 worms in each group. There is significant difference among these groups when symbols are different (P < 0.05). (**E**) The effects of *sod-3* RNAi and *hsp16.2* RNAi on DXN inhibiting worm paralysis induced by Aβ_1-42_ over-expression. Data are the average of three replicates with about 120–180 worms in each group. ***Indicated that there was significant difference between treatment groups and control group at P < 0.001.

DAF-16 is a crucial component of DAF-2 insulin like pathway to regulate many gene expressions for defending against various stresses including oxidative stress and proteotoxicity^33^, its activation has been proved to be benefit for delaying paralysis induced by human Aβ excessive expression in the muscle in *C. elegans* ^34^. To examine whether DAF-16 is required for DXN protecting worms from severe stress of aberrant Aβ generation and accumulation, we knocked down expression of the transcription factor DAF-16 by RNAi, and found that the inhibitory effect of DXN on worm paralysis significantly decreased (Fig. 7A). We further constructed a *daf-16* mutant strain under genetic background of the temperature sensitive Aβ strain CL4176 by standard genetic crosses. Treating the AD worms with *daf-16* mutation, a similar result has been obtained (Fig. 7B). These results indicated that DXN reduced Aβ toxicity at least partially through a DAF-16-based mechanism. After DXN treatment, DAF-16 was indeed activated and displayed nuclear translocation from the cytosol to the nucleus (Fig. 7C,D). Further, its typical downstream target SOD-3 was markedly up-regulated (Fig. 8A,B). According to our knowledge, DAF-16 activation increases stress resistance and then leads to lengthen lifespan. Expectedly, the result of lifespan assay on *unc-54*/human Aβ_1-42_ worm strain CL2006 treated with DXN showed that DXN did significantly extend worm lifespan (Fig. 3S).

As a chaperon, HSP-16.2 can be induced by the abnormal Aβ proteins to increase their sequestration, degradation, and refolding^35^. In our experiment, the expression of *hsp-16.2* was examined after DXN pretreatment by using TJ375. The transgenic worm strain TJ375 has a GFP reporter gene controlled by the promoter of *hsp-16.2*, it exhibits an excessive GFP expression in the pharynx after exposure to oxidative stress such as juglone. The results showed that DXN alone did not significantly affect the *hsp-16.2*::*gfp* expression. However, when *C. elegans* were pretreated with DXN and further administrated with juglone, the fluorescence density in the pharynx of worms under juglone-induced oxidative stress significantly decreased compared to the DXN untreated group (Fig. 8C,D). We further treated AD worms with RNAi for *sod-3* and *hsp-16.2* (Fig. 8E). The results showed that *sod-3* RNAi significantly promoted worm paralysis suggesting that SOD-3 is required for battling against Aβ toxicity. It is seemed to find that DXN delayed AD-like symptoms to the same degree as DXN treatment alone without *sod-3* RNAi, but it did not indicate that SOD-3 was not required for DXN-mediated neuroprotection. Possibly, the compensatory upregulation of other *sod* genes or related detoxifying signaling pathway counteracted the effect of *sod-3* RNAi on DXN-mediated delaying worm paralysis^36^. As for *hsp16.2* RNAi, no obvious effect was observed. It might be explained by that *hsp-16.2* is only expressed under stress^37^.

Transcription factor SKN-1 modulates genes that fight against oxidative and xenobiotic stress and genes that regulate protein homeostasis^33^. After *skn-1* RNAi, the Aβ-induced paralysis phenotype in the temperature sensitive Aβ strain CL4176 was still significantly delayed by DXN treatment (Fig. 1S-A), indicating that SKN-1 was not involved in the protective effect of DXN. It was further validated by SKN-1 did not appear nuclear translocation at all (Fig. 1S-B).

HSF-1 regulates many gene expressions in response to stress^38^, and has been recognized to control disaggregation and degradation of Aβ^34^. In present work, after *hsf-1* RNAi, the Aβ-induced paralysis phenotype in the temperature sensitive Aβ strain CL4176 was still significantly delayed by DXN treatment (Fig. 1S-C), indicating that HSF-1 was not involved in the protective effect of DXN. In present work, RNAi for *hsf-1* and *skn-1* significantly promoted worm thermal stress sensitivity (Fig. 1S-D). Therefore, the lack of effect of RNAi on worm paralysis after DXN treatment could not result from that the RNAi treatment did not work.

## Discussion

DXN has been clinically used to treat epilepsy in China since 1990s. In present study, it strongly ameliorated Aβ-induced paralysis and 5-HT hypersensitivity in the transgenic *C. elegans* in a dose dependent manner (Fig. 1). Furthermore, DXN significantly alleviated learning and memory impairment induced by scopolamine in mice (Fig. 2). Those results indicated that DXN was potential to serve as a drug candidate for treating AD. In fact, we also tested the anti-AD activity of other two traditional Chinese formulas of Dianxianping tablet (DXP) and Guishaozhenxian tablet (GS), these two formulas also have been approved by CFDA for treating epilepsy. Our results showed that DXP and GS are less effective in comparison to DXN at their concentration available (Fig. 2S), and both of them could not be used on a larger dosage due to that they have severely delayed worm growth and development at their concentration in present work. Therefore, although there is close link between epilepsy and AD, whether an anti-epileptic drug can be used for treating AD still needs to be carefully investigated in details.

In present work, Vj has been proved to exert its delaying paralysis of AD-like symptom as a principal component (Fig. 3A). Further, compounds of 1–15 isolated from Vj significantly delayed worm paralysis and have anti-AD activity on a certain degree, except that compound 15 did not affect worm paralysis and compound 11 even aggravated AD-like symptom. In comparison with the complete herb of Vj, they only exhibited moderate protective effect (Fig. 5). Similarly, coffee extract can protect worms from Aβ toxicity, whereas caffeine does only to a much lesser degree^19^. Previously, Vj has been demonstrated to have anti-inflammatory and anti-oxidant activity^14, 15^. Several other compounds of iridoids, sesquiterpenoids extracted from this species and other related species of genus *Valeriana* have been shown to have neuroprotective effects^25, 39^. It is reasonable to conclude that these compounds can exert additive or synergistic effect for anti-AD. Of course, it cannot be excluded that more active compound will be isolated from Vj in the future work.

DXN, as a kind of traditional Chinese medicine, is composed of eight herbs. Among the herbs, besides Vj, Ra and Ru have anti-AD activity^1, 14–17^. Our results show that Ra and Ru did not ameliorate AD-like symptom in worms additively or synergistically (Fig. 3A), but complete DXN did not delay nematode growth any more (Fig. 3B), indicating that DXN is less toxic than Vj alone. It is especially important for a potential anti-AD drug to act effectively to be used just at asymptomatic and preclinical stage, and the disease manifests its clinical symptoms after a decade or more, thus anti-AD therapy being a long term prevention^40^. The present evidence supported that DXN could not be reduced. Further work needs to investigate on the actions of these herbs through a feasible AD model *in vivo* or *in vitro* based on other respects of the pathogenesis of this devastating disease.

After DXN treatment, Aβ oligomers significantly reduced, but Aβ monomers increased in AD worms (Fig. 6C,D). It is well recognized that Aβ oligomers induce the death of neurons and are responsible for AD-related memory loss. Moreover, Aβ oligomers are closely related to severity of dementia in AD patients^1, 39^. Accumulating evidence suggests that Aβ oligomers but not Aβ monomers or Aβ deposits are correlated with Aβ toxicity^41, 42^. Our results supported that DXN strongly ameliorating Aβ toxicity in the transgenic *C. elegans* overexpressed human Aβ_1-42_ might be a consequence of its directly or indirectly promoting the shift from the toxic Aβ oligomer to less-toxic monomer form.

Small HSPs are Low-molecular-weight Heat Shock Proteins (12–43 kD) in response to a series of injuries including thermal stress and oxidative stress^43^. Moreover, small HSPs also participate in preventing the accumulation of several different types of toxic proteins such as Aβ and ployQ^44^. HSP16.2 can suppress Aβ toxicity by assisting abnormal protein sequestration, degradation, and refolding in AD *C. elegans* ^35^. Here, DXN treatment did not increase the expression of *hsp-16.2* (Fig. 8C,D), indicating that toxic Aβ oligomer reduction after DXN treatment was not mediated by molecular chaperon HSP-16.2. Then whether DXN can directly bind to Aβ proteins to inhibit their aggregation and increase their less toxic monomers needs further investigated. However, DXN counteracted the up-regulation of *hsp-16.2* induced by juglone (Fig. 8C,D). Since juglone is a reactive oxygen species generator^45^, it gave us a notion that DXN could reduce Aβ toxicity through anti-oxidant activity. It agrees with the previous work that components of DXN, Vj and *Rhizoma Acori tatarinowii* possess high antioxidant activity^14, 46, 47^.

**Figure 9:**
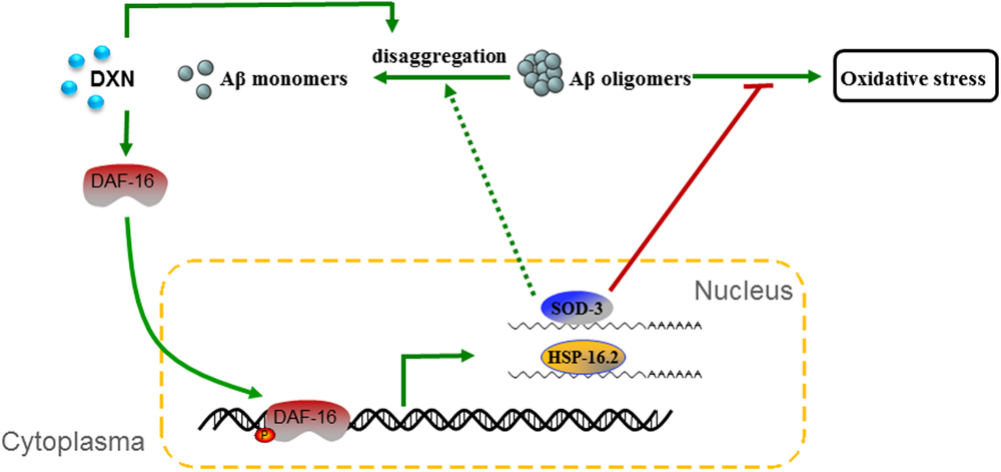
Schematic diagram of DXN action on suppressing Aβ toxicity. DXN directly or indirectly reduced Aβ oligomers and increased less toxic Aβ monomers, further, DXN induced DAF-16 activation to regulate its downstream stress responsive gene expressions, such as *sod-3*, *hsp-16.2*, at least partially to exert its anti-AD activity.

SOD-3 protects *C. elegans* from oxidative stress and participates in suppressing the Aβ toxicity^48, 49^. In present work, our results showed that DXN indeed promoted the expression of *sod-3* (Fig. 8A,B). It supported that DXN increased organism stress resistance and armed *C. elegans* to defend against oxidative stress induced by aberrant Aβ proteins^50^. SOD-3 locates downstream of DAF-16 in *C. elegans* ^﻿51, 52^. By turns, DAF-16 is a FOXO transcription factor and acts as a crucial component in the DAF-2/DAF-16 insulin like signaling pathway^34, 53^. The activation of DAF-16 will lead to elevated expression of genes in responses to a wide range of stressors^54, 55^. Our results showed that DXN could significantly activate DAF-16 to translocate from cytosol to nucleus (Fig. 7C,D), and DAF-16 is required for the delaying paralysis effect of DXN (Fig. 7A,B). HSF-1 and SKN-1 are transcriptional factors in parallel to DAF-16, which also can promote the expression of stress responsive genes to inhibit the toxicity of Aβ^33, 34, 38^. In our experiment, *hsf-1* and *skn-1* RNAi did not affect the DXN delaying paralysis of anti-AD action (Fig. 1SA,B). Additionally, SKN-1 did not exhibit nuclear translocation after DXN treatment (Fig. 1SC). In conclusion, as shown in Fig. 9, DXN suppressing Aβ-induced pathological behaviors was at least partially mediated by DAF-16 activation in worms. DXN also directly or indirectly reduced Aβ oligomers and increased less toxic Aβ monomers.

## Materials and Methods

### Preparation of DXN

DXN is composed of eight herb drugs at dose of 500 g *Valerianae jatamansi Rhizoma et Radix* (Vj), 200 g *Semen Pharbitidis*, 200 g *Ramulus Uncariae cum Uncis* (Ru), 500 g *Rhizoma Acori tatarinowii* (Ra), 200 g *Rhizoma et Radix Nardostachyos*, 15 g *Semen Euphorbiae*, 0.3 g Menthol, 0.62 g *Radix et Rhizoma Valeriana officinalis*. DXN was prepared according to WS3-B-2823-97 in Drug Standard issued by Ministry of public health, China (Fourteenth prescriptions of Chinese Medicine). Briefly, herb drugs were crushed into coarse powder and extracted by diacolation and decoction methods. Among them, 200 g Vj coarse powder without extraction and menthol were directly added into the mixtures of other herb drug extractions. Finally, the mixtures were crushed into 1000 tablets of DXN. Quality control of DXN was performed by HPLC^56^. Baldrinal was used as marker substance and its content was 0.930 mg/g DXN. Total iridoids and sesquiterpenoid were measured by ultraviolet spectrophotometry according to Li *et al*. (2016)^57^. Total iridoids and sesquiterpenoid were 22.7 mg/g DXN.

In present work, DXN was produced by Kunming Chinese Medicine Factory Co., Ltd. (License Number: H.M.L.N. Z53020771). It was pressed and grinded into powder with a pestle in a mortar, and then dissolved in sterile water, centrifuged at 10,000 rpm for 10 min, the resultant supernatant containing DXN at a concentration of 150 mg/mL as a stock solution was stored at 4 °C before use.

### *C. elegans* strains and maintenance

The wild-type *C. elegans* N2, transgenic *C. elegans* CL2355 [*snb-1*/Aβ1-42/long 3′-UTR + *mtl-2*::GFP]; CL2122 dvIs15 [(pPD30.38) unc-54(vector) + (pCL26) *mtl-2*::GFP]; CL4176 *smg-1*(cc546)I; dvIs27[*myo-3*p::Aβ(1-42)::*let-851* 3′UTR) + *rol-6*(su1006)]X; CL2006, dvIs2 [pCL12 (*unc-54*/human Aβ peptide1-42 minigene) + pRF4]; CL2179, *smg-1*[*myo-3*::GFP]; TJ356 [zIs356 (P*daf-16*:: *daf-16*a/b::GFP + *rol-6*)]; TJ375[gpIs1(*hsp-16.2*::GFP)]; CF1553 [muIs84 ((pAD76) *sod-3*p::GFP + *rol-6*)]; LG333[geIs7(*skn-1b*::GFP)]; CF1038 [*daf-16*(mu86) I] were obtained from Caenorhabditis Genetics Center (CGC) (University of Minnesota, Minneapolis, MN). All worms were propagated at 20 °C except CL4176 at 16 °C on solid nematode growth medium (NGM) seeded with standard food resource of *E. coli* OP50.

### Paralysis assay

Transgenic *C. elegans* of temperature sensitive Aβ strain (CL4176) maintained at 16 °C were egg-synchronized onto the NGM plates containing with 0, 1 mg/mL, 5 mg/mL, 15 mg/mL DXN. Other principal herbs of Vj, Ra and Ru were used at an equivalent dose as they were in DXN, and 3.2 mg/mL Vj, 3.7 mg/mL Ra and 0.6 mg/mL Ru was used, respectively. For compounds of 1–15 extracted from Vj, they were all diluted at indicated final concentrations in 0.1%DMSO according to their maximum saturated solubility. Compounds of 1–7 were relatively used at a final concentration of 50 μM, 100 μM, 20 μM, 20 μM, 20 μM, 160 μM, 120 μM, compounds of 8–11 were used at 250 μM and compounds of 12–15 were used at 100 μM. Worms were induced to express human Aβ_1-42_ till they were at L3 stage larvae by up-shifting the incubation temperature from 16 °C to 25 °C for 34 h. The transgenic worms were scored at 2 h intervals for paralysis till all animals in negative control group were paralyzed. Each worm was gently touched with a platinum loop to identify as the paralysis, nematodes were considered to be paralyzed if they did not move at all or only moved their heads^58^.

### Fluorescence staining of Aβ deposits

Transgenic *C. elegans* with *unc-54*/human Aβ_1-42_ maintained at 20 °C were egg-synchronized onto NGM plates and grown until the L4 stage, worms were then moved to fresh NGM plates containing 0, 1 mg/mL, 5 mg/mL, 15 mg/mL DXN. Two days later, Thioflavine-S (ThS) staining was performed as described previously^22^. Briefly, worms were collected by washing with M9 and were fixed in 4% paraformaldehyde in PBS, pH7.4, at 4 °C for 24 h. The fixative solution was replaced by permeabilization solution (5% fresh β-mercaptoethanol, 1% Triton X-100, 125 mM Tris-HCl, pH7.4), and incubated in a 37 °C incubator for another 24 h. The animals were washed three times in PBS-T (PBS plus 0.1% Triton X-100), stained with 0.125% ThS (Sigma) in 50% ethanol for 2 min, and the samples were destained with 50% ethanol twice for 2 min. Stained samples were resuspended in 100 μL PBS, and finally mounted and observed by fluorescence microscopy (BX53; Olympus, Japan). Amyloid deposits in the anterior area of each animal were quantified by scoring the number of ThS reactive deposits. *C. elegans* N2 were used as amyloid deposit control.

### Exogenous serotonin sensitivity assay

After egg-synchronized, the transgenic *snb-1*/Aβ_1-42_ worm were placed at 20 °C on NGM plates seeded with OP50 for 48 h. The worms were treated with 0, 1 mg/mL, 5 mg/mL, 15 mg/mL DXN for another 48 h, respectively. 30 worms in each group were washed with M9 buffer for three times and were transferred into 200 μL M9 buffer containing 1 mg serotonin (Sigma), paralyzed worms were scored after 5 min. Animals were considered to be paralyzed if they did not move at all within 5 sec. Worm strain CL2122 without Aβ expression in nerve cells was used as a transgenic control.

### Western blotting

Human Aβ_1-42_ in the temperature sensitive Aβ strain (CL4176) was identified by immunoblotting on a Tris-Tricine gel by the standard Western blotting assay. Wild type N2 was used as negative control of the expression of Aβ. Worms were treated as paralysis assay with or without 15 mg/mL DXN. Nematodes were then washed three times in M9 to remove the bacteria after incubation temperature up-shifting for 40 h and finally harvested in distilled water with protease inhibitor cocktail (Sigma, P2714) added, quickly frozen in liquid nitrogen and stored at -80 °C. Worms were boiled in sample lysis buffer (1× protease inhibitor cocktail, 62 mM Tris-HCl, pH 6.8, 2% SDS, 10% glycerol, 4% β-mercaptoethanol) for 10 min, then seated on ice to cool, centrifuged at 14,000 g for 5 min. Total protein content in the supernatant was measured by Lowry method. Proteins were boiled for 5 min in sample loading buffer (62 mM Tris-HCl, pH6.8, 2% SDS, 10% glycerol, 4% β-mercaptoethanol, 0.0005% bromophenol blue). Samples were performed electrophoresis on the Tris-Tricine gel with 70 μg total protein in each lane. After the gel was transferred to 0.22μm PVDF membrane, the membrane was boiled in PBS for 15 min before blocking with a solution of 5% milk in TBS-Tween (100 mM Tris-HCl, pH7.5, 150 mM NaCl, 0.1% Tween-20) for 1 h at room temperature. Blotting was carried out overnight at 4 °C. Aβ proteins were detected by 6E10 (Biolegend, 803001) at 1:1000 dilution. Internal control of α-tubulin was probed by polyclonal antibody against it (Sigma, T6199) at 1:1000 dilution. Goat anti-mouse HRP-labelled IgG was used as a secondary antibody.

### Nuclear localization of DAF-16

Worm *daf-16*::*gfp* (TJ356) expressing GFP as a reporter was used for detecting the intracellular distribution of DAF-16^59^. Synchronized L1 larvae worms were fed with or without 15 mg/mL DXN for 72 h. Subsequent to this treatment, worms were mounted on glass slides and their nuclear localization of DAF-16 were observed under a fluorescence microscope (BX53; Olympus, Japan). Worms are scored based on two categories of cytosolic and nuclear distribution with respect to the major localization of the DAF-16::GFP fusion protein. DAF-16 intracellular location was indicated by the ratio of nuclear translocation in the experimental population.

### RNA interference (RNAi)

RNAi gene expression clones were constructed as described by Fraser *et al*.^60^. Briefly, PCR products were synthesized using Q5 High-Fidelity DNA Polymerase (New England Biolabs, USA) with genomic DNA as template by relative primers listed in Table 2, and then inserted into L4440 vector (Addgene plasmid 1654). Recombinant plasmids were subsequently transformed into the *E. coli* HT115 (DE3) bacterial strain using a standard method. The desired positive colonies were verified by sequencing. The sequences of target genes were available from the web site http://www.wormbase.org/#01-23-6.

**Table 2:** List of primers for amplifying target genes.

Gene	Primer name	Primer sequence	Restriction enzyme
*daf-16*	daf-16-F	5′ AAAACTGCAGAGTACAGCAATTCCCAAATGAAA 3′	Pst I
	daf-16-R	5′ CCCAAGCTTAATTGGATTTCGAAGAAGTGGAT 3′	Hind III
*skn-1*	skn-1-F	5′ AAAACTGCAGGGGTACCCACTTGCCCTATT 3′	Pst I
	skn-1-R	5′CCCAAGCTTTCATTTCAGCCACTCACTGC 3′	Hind III
*hsf-1*	hsf-1-F	5′AAAACTGCAGGAAAAAAAGTAGGAGCAAAAAAT3′	Pst I
	hsf-1-R	5′CGGGGTACCAGTCAAAAAGCTGAAAAAATCGG3′	Kpn I
*hsp16.2*	hsp16.2-F	5′ CGGGGTACC ATTCAGCAGATTTCTCTTCGACGATT 3′	Kpn I
	hsp16.2-R	5′ CCGCTCGAGTGTCACTTTACCACTATTTCCGTCC 3′	Xho I
*sod-3*	sod-3-F	5′ CGGGGTACCAGCTCCTTTTAAATTAAGACA 3′	Kpn I
	sod-3-R	5′ CCGCTCGAGTATTCTTCCAGTTGGCAAT 3′	Xho I

HT115 (DE3) bacteria containing the target genes and expressing double stranded RNAs (dsRNA) that inactivate specified genes were used as previously described^58, 61^. Target gene expressions were knocked down by feeding the worms with dsRNA-containing *E. coli* HT115 strains from egg to adulthood at 16 °C. Except for *skn-1*, worms were exposed to RNAi bacteria from L3 stage larvae. The next generation of these worms were transferred to another RNAi plates and used for paralysis assay as above.

### Quantification of P*sod-3*::*gfp* expression via fluorescence microscopy

The transgenic *C. elegans* strain CF1553 expressing GFP as a reporter for inducible *sod-3* expression was used in our study. Age-synchronized L1 stage transgenic *C. elegans* were treated with or without 15 mg/mL DXN for 72 h, the expression of *sod-3*::*gfp* was evaluated through measuring the GFP fluorescence intensity of each whole worm. At least 25 randomly selected worms from each group were observed and photographed by fluorescence microscopy on a glass slide. To quantitatively measure the GFP fluorescence intensity in each whole worm, the Image J software (NIH, Bethesda, MD, USA) was used.

### Quantification of P*hsp-16.2*::*gfp* expression via fluorescence microscopy

In the transgenic strain TJ375, GFP is fused to *hsp-16.2* promoter for reporting the expression of HSP-16.2. Age-synchronized L1 stage P*hsp-16.2*::*gfp* worms were treated with or without 15 mg/mL DXN for 48 h, followed by exposure to 20 mM juglone for 24 h. The expression of *hsp-16.2*::*gfp* was evaluated through measuring the fluorescence intensity of the GFP reporter. At least 25 randomly selected worms from each group were measured by fluorescence microscopy on a glass slide. To quantitatively measure the GFP fluorescence intensity in the area anterior of the pharyngeal bulb in individual worms, Image J software (NIH, Bethesda, MD, USA) was used.

### Morris water maze test

The Morris water maze test was performed as described by Morris (1981)^62^. The apparatus was a circular pool of 120 cm diameter filled with water, and maintained at 22.0 ± 0.5 °C by a calefaction stick. A transparent platform of 11 cm diameter 1 cm below the water surface was placed at a fixed point of one quadrant. 4-6 weeks old Kunming mice were provided by the Experiment Animal Center of Lanzhou University. Animals were treated with DXN at dose of 0.39 g/kg and 2.34 g/kg by i.g. for 21 days, mice in positive control group administrated piracetam at dose of 0.5 g/kg by i.g. Amnesia was induced by scopolamine at dose of 3 mg/kg by i.p. 30 min after DXN or piracetam administration. Animals in control group were only received normal saline by i.g.. One day prior to the experiment, animals were trained to swim for 60 sec in the absence of the platform. Mice were performed single trial per day for consecutive 5 days, and escape latency of the time mice taken to swim to the platform was recorded by an automated tracking system (Ethovision XT software). 24 h after the experiment, a probe trial was performed to test for spatial memory.

All experiments were approved by the Animal Care and Use Committee of Lanzhou University, and the methods were carried out in accordance to the Guidance Suggestions for the Care and Use of Laboratory Animals, formulated by the Ministry of Science and Technology of China.

### Statistical analysis

Statistical analyses were performed by SPSS 17.0 software. The statistical significances of the results were analyzed by using one-way analysis of variance (ANOVA). Except for paralysis assays, log rank survival test was carried out to compare the significance level among treatments. The P value of 0.05 or less was considered to be significant statistically.

## Electronic supplementary material

Supplementary information
