# Supplementary material for: Dianxianning improved amyloid β-induced pathological characteristics partially through DAF-2/DAF-16 insulin like pathway in transgenic C. elegans
Source: Sci Rep. 2017 Sep 12;7:11408. doi: 10.1038/s41598-017-11628-9 (PMC5595840; doi:10.1038/s41598-017-11628-9)
Supplement: Supplementary file 1 — Supplementary information [file 41598_2017_11628_MOESM1_ESM.doc]

**Dianxianning improved amyloid β-induced pathological characteristics partially through DAF-2/DAF-16 insulin like pathway in transgenic *C. elegans***

### Dejuan Zhi, Dong Wang, Wenqi Yang, Ziyun Duan, Shuqian Zhu, Dong Juan, Na Wang,

### Ningbo Wang, Dongqing Fei, Zhanxin Zhang, Xin Wang, Meizhu Wang, Hongyu Li *

Gansu high throughput screening and creation center for health products, School of Pharmacy, Lanzhou University, Lanzhou, P.R. China

*Corresponding Author:

Lanzhou University, No. 199, Donggang West Road, Lanzhou 730000, P. R. China

Tel: 86-931-8915685; FAX: 86-931-8915685

Email: lihy@lzu.edu.cn

**Support information**

Nuclear localization of SKN-1

LG333 (*skn-1b*::GFP) expressing GFP as a reporter were used for detecting the intracellular distribution of SKN-1^1^. Drug treatment and nuclear localization of SKN-1::GFP fusion protein were observed as DAF-16 nuclear localization assay.

Lifespan assay

Synchronized *unc-54*/human Aβ1-42 worms were grown on NGM plates to L4 larvae, and then transferred onto fresh NGM plates containing 1mg/mL, 5mg/mL, 15mg/mL DXN, respectively. Worms were moved to such fresh NGM plates every other day to avoid overlapping generations till the end of their reproduction period. Since animals were treated with DXN, they were observed and scored as live or dead every day under a dissecting microscope till all animals were dead.

Reference:

1. An, J.H., Blackwell, T.K. SKN-1 links *C. elegans* mesendodermal specification to a conserved oxidative stress response. Gene Dev. 17, 1882-1893 (2003).

Figure 1S DXN inhibiting worm paralysis induced by Aβ over-expression was independent on HSF-1 and SKN-1. A: DXN anti-AD activity was not affected by *skn-1* RNAi. Data are the average of three replicates with 180 worms in each group. *** indicated that there was significant difference between treatment group and control group at P<0.001. B: SKN-1 was not activated after treatment with 15mg/mL DXN, and worms treated with 0.1 mM PQ were used as a positive control. Data are the average of three replicates with about 90 worms in each group. The scale bar was 20 μm. C: DXN anti-AD activity was not affected by *hsf-1* RNAi. Data are the average of three replicates with 180 worms in each group. *** indicated that there was significant difference between treatment group and control group at P<0.001. D: Compared to L4440 control group, the mortality rates of worms treated by *skn-1* RNAi and *hsf-1* RNAi markedly increased under thermal stress at 35℃ for 5.5h. Data are the average of three replicates with 180 worms in each group. There is significant difference among these groups when symbols are different (P<0.05).

Figure 2S The effects of DXP and GS on AD-like symptoms in Aβ transgenic *C. elegans*. DXP and GS are also anti-epilepsy drugs, which are approved by CFDA and recorded in Chinese Pharmacopoeia (2015 Edition). Both of them showed significant anti-AD activity, but on much lesser degree in comparison with DXN. It is deserved to notice that DXP and GS were within their permissible concentrations, so that they did not delay worm growth and development. Data are the average of three replicates with about 180 worms in each group. *** indicated that there was significant difference between treatment group and control group at P<0.001.

Figure 3S The effects of DXN on lifespan of *unc-54*/human Aβ1-42 worms. Data are the average of three replicates with about 100 worms in each group.


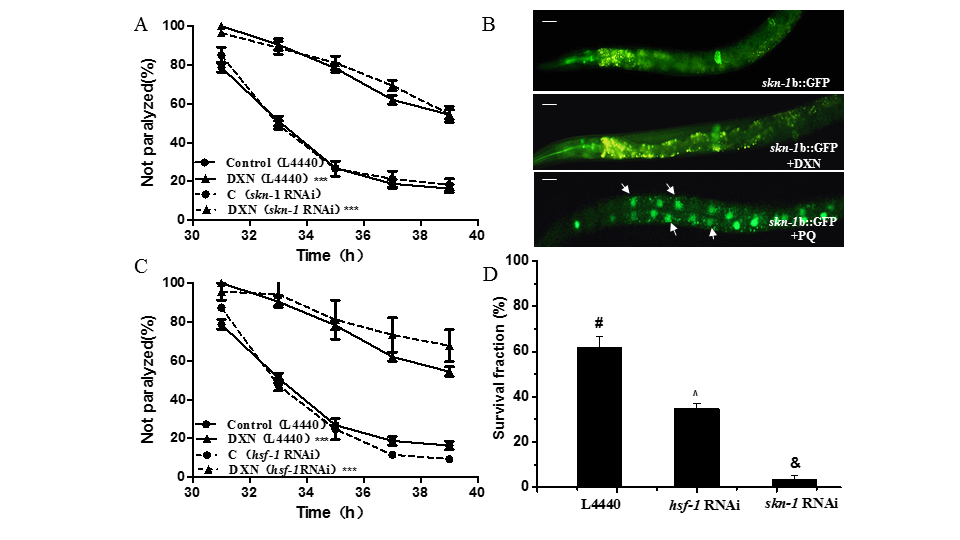


Figure 1S


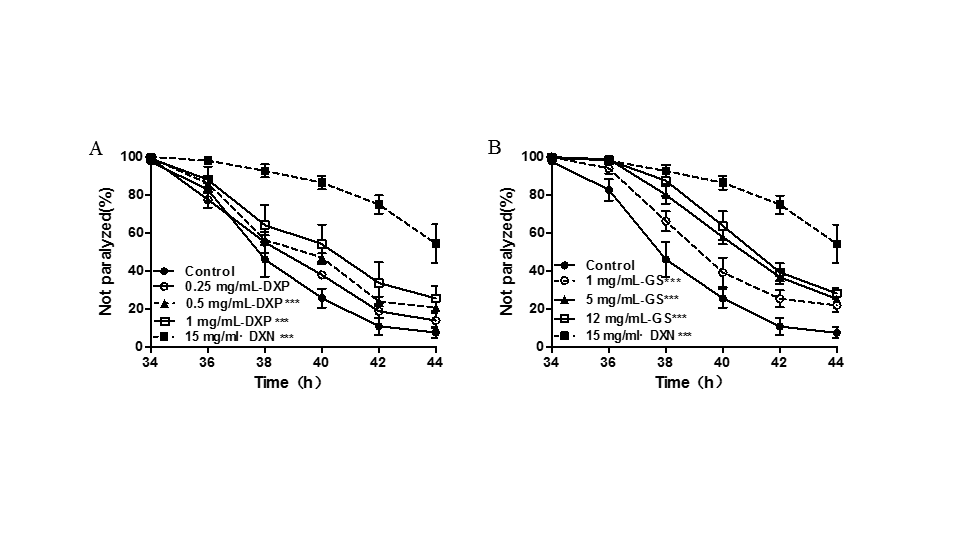


Figure2S


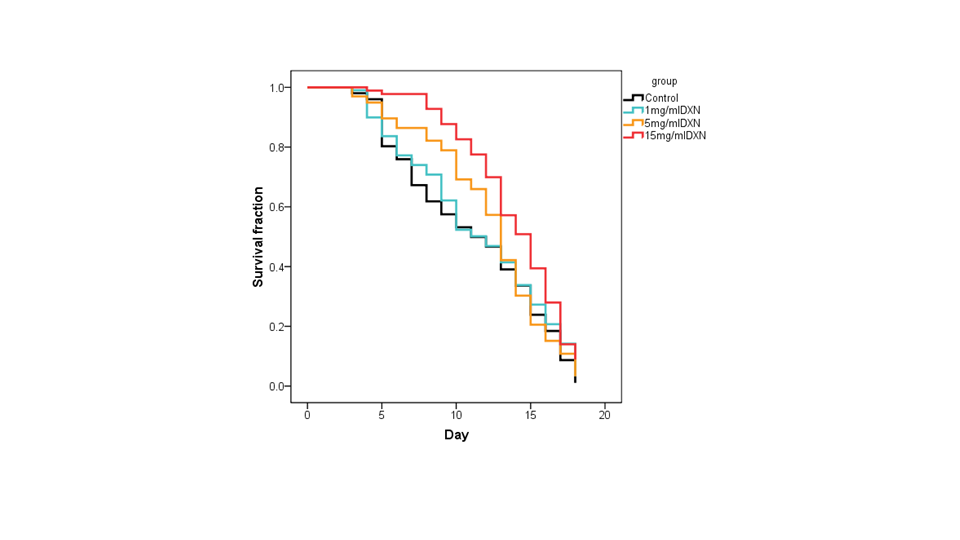


Figure3S
